# An olfactory-prefrontal cortical circuit supports social recognition

**DOI:** 10.21203/rs.3.rs-9613537/v1

**Published:** 2026-06-01

**Authors:** Janardhan P. Bhattarai, Kun Yang, Jingqi He, Geronimo Velazquez-Hernandez, Yingqi Wang, Yun-Feng Zhang, Abby G. Lieberman, Omer Zeliger, Tammi Coleman, Yina Zhou, Megan Tu, Brittany C. Chapman, Krishna Jaladanki, David Kedeme, Atsushi Kamiya, Andreia V. Faria, Wenqin Luo, Akira Sawa, Minghong Ma

**Affiliations:** 1 Department of Neuroscience, University of Pennsylvania Perelman School of Medicine, Philadelphia, PA, USA; 2 Department of Psychiatry, Johns Hopkins University School of Medicine, Baltimore, MD, USA; 3 Department of Radiology, Johns Hopkins University School of Medicine, Baltimore, MD, USA; 4 Department of Neuroscience, Johns Hopkins University School of Medicine, Baltimore, MD, USA.; 5 Department of Biomedical Engineering, Johns Hopkins University School of Medicine, Baltimore, MD, USA; 6 Department of Pharmacology, Johns Hopkins University School of Medicine, Baltimore, MD, USA; 7 Department of Genetic Medicine, Johns Hopkins University School of Medicine, Baltimore, MD, USA; 8 Department of Mental Health, Johns Hopkins Bloomberg School of Public Health, Baltimore, MD, USA.

**Keywords:** anterior olfactory nucleus, medial prefrontal cortex, social recognition, NMBR-Cre mice, resting-state fMRI

## Abstract

Social recognition, the ability to distinguish between individuals, is essential for cognitively demanding social behaviors. The anterior olfactory nucleus (AON), a primary olfactory cortical region, is implicated in this process, but the underlying neurocircuitry remains poorly understood. Here, we generated a novel mouse line to enable genetic access to AON pyramidal neurons and mapped their whole-brain synaptic inputs and outputs. The medial prefrontal cortex (mPFC), a crucial hub for social cognition, is the primary neocortical target of AON neurons, which form monosynaptic excitatory connections with a substantial fraction of mPFC neurons. The AON→mPFC pathway is activated during social investigation, and chemogenetic inhibition of this pathway impairs social recognition. Moreover, an analogous AON-prefrontal pathway is present in humans, as supported by resting-state functional magnetic resonance imaging (fMRI) functional connectivity analyses. Taken together, these findings reveal a conserved olfactory-prefrontal circuit spanning mice to humans, potentially linking olfactory dysfunction to neuropsychiatric disorders.

## Introduction

Social species gain numerous evolutionary advantages that enhance survival and adaptability. Sociability (inclination to interact with conspecifics) and social recognition (the ability to distinguish among individuals within the same species) are integral components of social cognition, which is essential for individual well-being and societal functioning. Disruptions in social cognition are observed across a broad range of neuropsychiatric disorders^1,2^.

In rodents, chemosensory cues emitted by conspecifics from a variety of sources (e.g., urine, feces, skin, and glands) play critical roles in regulating social behaviors^3^. Social cues processed through the accessory olfactory system primarily mediate innate, species-specific social behaviors (reproduction, aggression, and maternal care), whereas the main olfactory system contributes to social cognition^4–7^. The main olfactory bulb (MOB), which receives direct input from olfactory sensory neurons in the olfactory epithelium, relays both smell and nasal breathing information to multiple olfactory cortical regions, including the piriform cortex (PIR) and anterior olfactory nucleus/tenia tecta (AON for simplicity)^6,8^. Converging evidence highlights a critical role for the AON in social cognition. The AON receives direct projections from the ventral hippocampus, a pathway implicated in odor memory and social recognition^9,10^. In addition, oxytocin released from the hypothalamus facilitates social recognition through oxytocin receptors expressed on AON neurons, and deletion of these receptors impairs social recognition^11,12^.

However, the downstream targets of the AON that mediate social cognition remain largely undefined. Neural circuit tracing studies demonstrate that AON neurons project directly to the medial prefrontal cortex (mPFC), including both the infralimbic and prelimbic cortices^13–16^. Extensive evidence identifies the mPFC and its associated circuitry are key regulators of social cognition^17–19^. The neuronal ensembles with the mPFC dynamically encode real-time social behavioral information^20–23^. Moreover, selective manipulation of specific mPFC neuronal subtypes and/or projection-defined pathways modulates social interaction, social recognition, and social dominance^24–28^.

Neural activity in the mPFC is strongly modulated by olfactory input. Beyond odor detection, the main olfactory system also conveys nasal breathing signals to the brain^29–32^, generating respiration-entrained rhythmic activity across widespread regions, including the mPFC^33,34^. Bulbectomy or ablation of the olfactory epithelium markedly reduces respiration-related activity in the mPFC^13,16,35,36^. Rodents characteristically alter their breathing and sniffing patterns during odor investigation^37,38^. It is plausible that during social behaviors, both odor-induced and respiration-coupled olfactory signals influence mPFC activity. However, the extent to which olfactory input shapes mPFC neuronal activity, and how the AON→mPFC circuit contributes to social cognition, remains unclear.

In this study, we generated a neuromedin B receptor (*Nmbr*)-Cre knock-in mouse line using CRISPR-Cas9-mediated gene editing, enabling genetic access to excitatory neurons in the AON. Whole-brain tracing of synaptic outputs of AON neurons identified the mPFC as the principal neocortical target. Patch-clamp recordings combined with optogenetics in acute brain slices demonstrated that AON neurons provide monosynaptic excitatory input to a substantial fraction of pyramidal neurons and interneurons in the infralimbic and prelimbic cortices. Using Ca^2+^-based fiber photometry, we further showed that AON neurons are engaged during multiple behaviors, including social investigation. Chemogenetic inhibition of the AON→mPFC pathway selectively impaired social recognition, without affecting sociability, in the three-chamber assay. Together, these findings uncover an olfactory-prefrontal circuit that contributes to social recognition. Finally, resting-state fMRI functional connectivity analyses support the presence of a homologous AON-prefrontal pathway in humans. Strong olfactory-prefrontal network coupling may therefore provide a neural substrate linking olfactory dysfunction to neuropsychiatric disorders.

## Results

### Targeting AON neurons in *Nmbr*-Cre mice

**Figure1.**
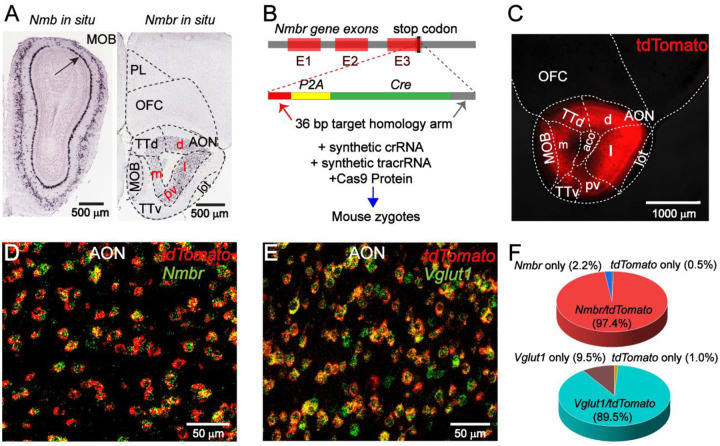
Generation and characterization of *Nmbr*-Cre knock-in mouse line. **(A)** *Nmb* and *Nmbr in situ* hybridization data from the Allen Brain gene expression atlas^76^. **(B)** Strategy for CRISPR-Cas9-mediated knock-in of P2A-Cre into the 3’UTR of the *Nmbr* gene (see Methods for details). **(C)** Coronal section from an *Nmbr*-Cre*;tdTomato*^f/+^ mouse showing tdTomato+ neurons in the AON. **(D, E)** RNAscope multiplex fluorescent assays for *Nmbr* vs *tdTomato* (D) and *tdTomato* vs *Vglut1* (E) in the AON. **(F)** Quantification of co-labeled AON neurons (N=3 mice; 3 sections/mouse).

To gain genetic access to AON neurons, we conducted a targeted differential gene expression search within the Allen Brain Atlas mouse *in situ* hybridization database. We found that the neuromedin B receptor gene (*Nmbr*) is highly expressed in the AON but not in the nearby neocortex (Fig. 1A right panel). Coincidentally, *Nmb* is highly expressed in the MOB, presumably in the projection neurons, situated in the mitral cell layer and glomerular layer (Fig. 1A left panel). Using the CRISPR-Cas9 gene-editing technique^39,40^, we generated an *Nmbr*-Cre knock-in mouse line (Fig. 1B; see Methods for details). We then crossed *Nmbr*-Cre mice with the *tdTomato*^f/+^ reporter Ai9 line and obtained *Nmbr*-Cre*;tdTomato*^f/+^ mice (Fig. 1C). Consistent with the *in-situ* hybridization data (Fig. 1A), we found that tdTomato is highly expressed in the AON but not in the surrounding neocortex (Fig. 1C). Using the RNAscope multiplex fluorescent assay^41^, we found that *tdTomato*+ and *Nmbr*+ are always co-expressed in AON neurons (Fig. 1D), confirming that *Nmbr*-Cre mice recapitulate endogenous *Nmbr* expression in the AON. Double staining between *tdTomato* and *Vglut1* revealed that nearly 90% *Vglut1*+ neurons are *NMBR/tdTomato*+ (Fig. 1E), suggesting that *Nmbr*-Cre mice allow genetic access to a large portion of AON glutamatergic neurons.

### Whole-brain tracing of synaptic outputs and inputs of AON neurons

**Figure 2.**
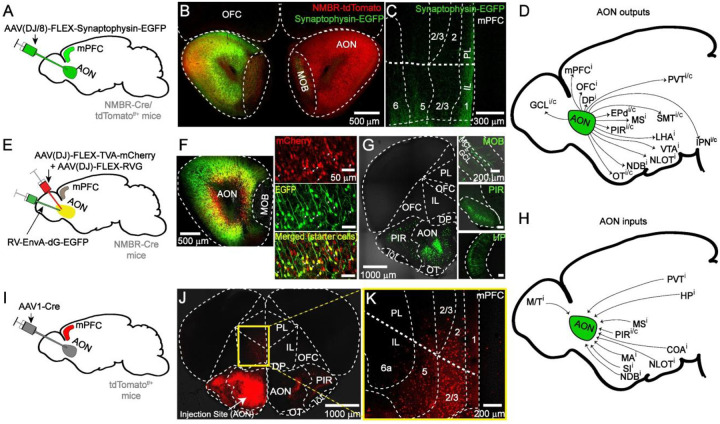
Whole-brain tracing of synaptic inputs and outputs of AON neurons. **(A)** Schematic of viral injection for anterograde tracing from AON neurons. **(B)** Confocal image showing EGFP expression in the injection site on one side of the AON. **(C)** EGFP-expressing axon terminals of AON neurons in the ipsilateral mPFC. **(D)** Summary of postsynaptic targets of AON neurons (N=5 mice). **(E)** Schematic of retrograde monosynaptic tracing via helper AAVs and pseudotyped-rabies virus injection in AON. **(F)** Left, confocal image showing the viral injection site in the AON. Right, expression of TVA-mCherry receptor and pseudotyped RV-EGFP double-positive starter cells in the AON. **(G)** Retrogradely labeled presynaptic partners of AON neurons in various brain regions. **(H)** Summary of presynaptic inputs to AON neurons (N=3 mice). **(I)** Schematic of viral injection for anterograde trans-synaptic tracing. **(J)** Confocal image showing the viral injection site in the AON (white arrow). **(K)** Trans-synaptically labeled tdTomato^+^ neurons in different layers of the mPFC. Similar results were obtained from 3 mice. In D and H, i = ipsilateral. c = contralateral. MOB, main olfactory bulb. OFC, orbitofrontal cortex. PL, prelimbic. IL, infralimbic. PIR, piriform cortex. HP, hippocampus. DP, dorsopeduncular cortex. GCL, granule cell layer of the MOB. MCL, mitral cell layer of the MOB. M/T, mitral/tufted cells of the MOB. PVT, paraventricular nucleus of thalamus. EPd, dorsal endopiriform nucleus. MS, medial septum. SMT, submedial nucleus of thalamus. LHA, lateral hypothalamus. IPN, interpeduncular nucleus. NLOT, nucleus of the lateral olfactory tract. NDB, nucleus of the diagonal band. OT, olfactory tubercle. COA, cortical nucleus of the amygdala. MA, Magnocellular nucleus. SI, Substantia innominata.

To dissect out the neural circuitry in which the AON exerts its functions, we traced whole-brain postsynaptic targets of AON neurons by unilaterally injecting AAV(DJ/8)-FLEX-synaptophysin-EGFP virus into the AON of *Nmbr*-Cre*;tdTomato*^f/+^ mice (Fig. 2A). AON neurons project to various cortical and subcortical regions (Supplemental Fig. S1 and Fig. 2D). Among the neocortical regions, AON neurons only project to the prefrontal cortex, and the mPFC is the major target. The axonal terminals of AON neurons can be visualized in the ipsilateral mPFC, densely innervating the infralimbic cortex (layer 1, 5, and 6) and thinning out towards the prelimbic and anterior cingulate cortex (Fig. 2B–2C).

We next characterized whole-brain monosynaptic inputs into AON neurons via pseudotyped rabies virus (RV) tracing^42,43^. We unilaterally injected Cre-dependent AAVs that bicistronically express TVA-mCherry, a required receptor for EnvA-pseudotyped RV, and RV glycoprotein, required for transsynaptic spread, into the AON of NMBR-Cre mice. Ten days later, EnvA-pseudotyped RV-EGFP virus was injected into the same AON site (Fig. 2E). Brains were perfused and fixed 7 days post-RV injection and sectioned coronally (100 μm thickness) for confocal microscopy imaging. We first verified that the “yellow” neurons (TVA-mCherry and RV-EGFP double-positive; presumptive “starter” cells) were located within the AON (Fig. 2F). As expected from the known neuronal projections, we found presynaptic cells (EGFP^+^ only) in the olfactory areas and the ventral hippocampus, but not in the mPFC (Fig. 2G, H), indicating that the AON to mPFC projection is unidirectional.

To locate the cell bodies of mPFC neurons that receive direct AON projection, we took advantage of the anterograde transsynaptic feature of the AAV1-Cre virus by unilaterally injecting it into the AON of the Ai9 *tdTomato*^f/+^ reporter mice (Fig. 2I, 2J). This strategy works well because the AON→mPFC projection is unidirectional. Consistent with the axonal projection pattern (Fig. 2C), we found more tdTomato^+^ cells in the infralimbic cortex (predominantly in layer 2/3 and 5) than in the prelimbic cortex (Fig. 2K).

### Monosynaptic connections from AON to mPFC neurons

**Figure 3.**
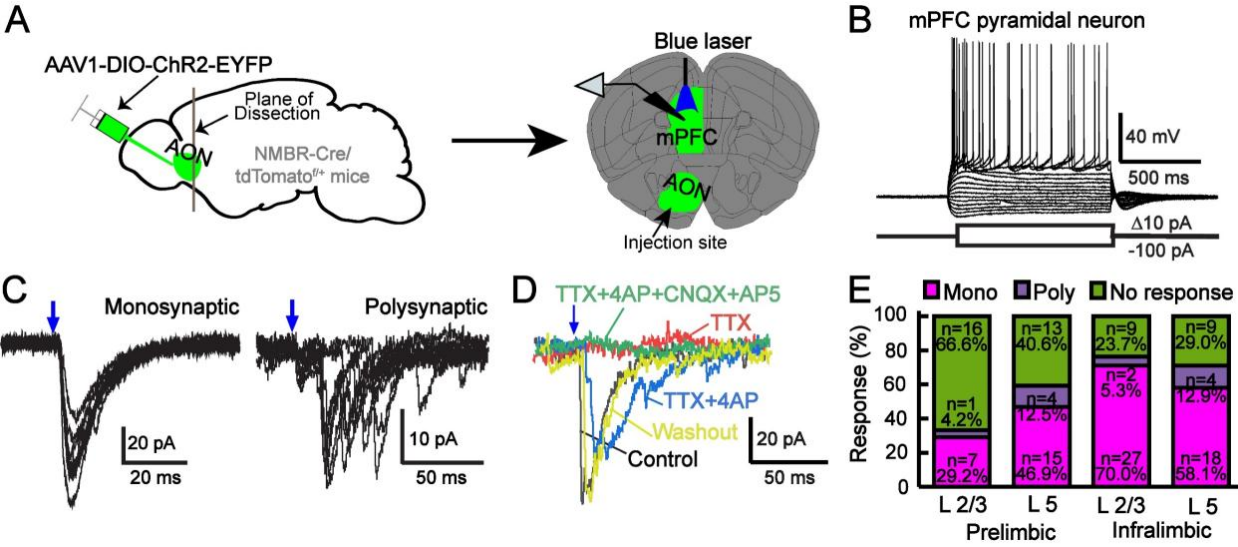
AON neurons make excitatory synaptic connections onto mPFC pyramidal neurons. **(A)** Schematic of viral injection and patch-clamp recording combined with optogenetics in slices. The coronal section image and those in Figs. S2A and 4A are from the Scalable Brain Atlas^77^. **(B)** Firing pattern of mPFC pyramidal neurons upon current injection. The baseline membrane potential was kept at −70 mV under current-clamp mode. **(C)** Blue light stimulation of ChR2-EYFP^+^ fibers of AON neurons in mPFC induced mono- and poly-synaptic excitatory postsynaptic currents on mPFC pyramidal neurons. **(D)** Light evoked responses with short latency were blocked by TTX (1 μM), revived by TTX+4AP (1 mM), and further blocked by CNQX (20 μM)+AP5 (50 μM). Similar results were obtained from 5 cells. **(E)** Summary of synaptic responses in L2/3 and L5 prelimbic and infralimbic pyramidal neurons upon blue light activation of AON neuron axons. Voltage-clamp mode with holding potential = −70 mV. Light pulse = 1 ms. Data were obtained from 15 mice.

We examined the synaptic properties of the AON→mPFC projection by combining patch clamp recording and optogenetics. Cre-dependent AAV1-DIO-ChR2-EYFP virus was unilaterally injected into the AON of *Nmbr*-Cre mice to drive Channelrhodopsin (ChR2) expression in AON neurons (Fig. 3A). Four weeks later, the injection site (AON) was verified by EYFP^+^ cells and acute brain slices containing the mPFC were prepared. We recorded presumptive pyramidal neurons (based on their soma size and shape) and verified their regular firing patterns upon current injections (Fig. 3B). We focused on the infralimbic and prelimbic cortices, as the anterior cingulate cortex contained very few fibers and only sparse anterogradely transsynaptically labeled neurons from the AON. Under voltage-clamp mode, blue light evoked excitatory postsynaptic currents (EPSCs) in mPFC neurons from different layers (Fig. 3C). Monosynaptic connectivity was verified by short latency (< 6 ms) and little jitter (< 1 ms) upon repeated light stimuli and by pharmacological experiments in a subset of neurons (Fig. 3C). Tetrodotoxin (TTX, 1 μM; a sodium channel blocker) blocked action potentials and abolished evoked EPSCs. Co-application of TTX and 4-AP (4-aminopyridine, 1 mM; a potassium channel blocker) enabled action potential-independent, ChR2-mediated neurotransmitter release. The reappearance of light-evoked EPSCs supports the presence of a monosynaptic connection. These EPSCs were completely blocked by glutamate receptor antagonists (CNQX, 20 μM and AP5, 50 μM) (Fig. 3D). Consistent with the above tracing results, there are more pyramidal neurons showing light-evoked monosynaptic EPSCs in the infralimbic cortex than in the prelimbic cortex (Fig. 3E).

To test whether AON neurons make synaptic connections onto mPFC interneurons, we conducted similar experiments in *Vglut1*-Cre;*tdTomato*^f/+^ mice, in which we could target interneurons (*Vglut1*-tdTomato^−^ cells) in the mPFC (Supplemental Fig. S2). Similar to pyramidal neurons, a large portion of mPFC interneurons from layers 2/3 and 5 of infralimbic and prelimbic cortex exhibited light-evoked EPSCs and the majority of the connections were monosynaptic (Supplemental Fig. S2).

**Figure 4.**
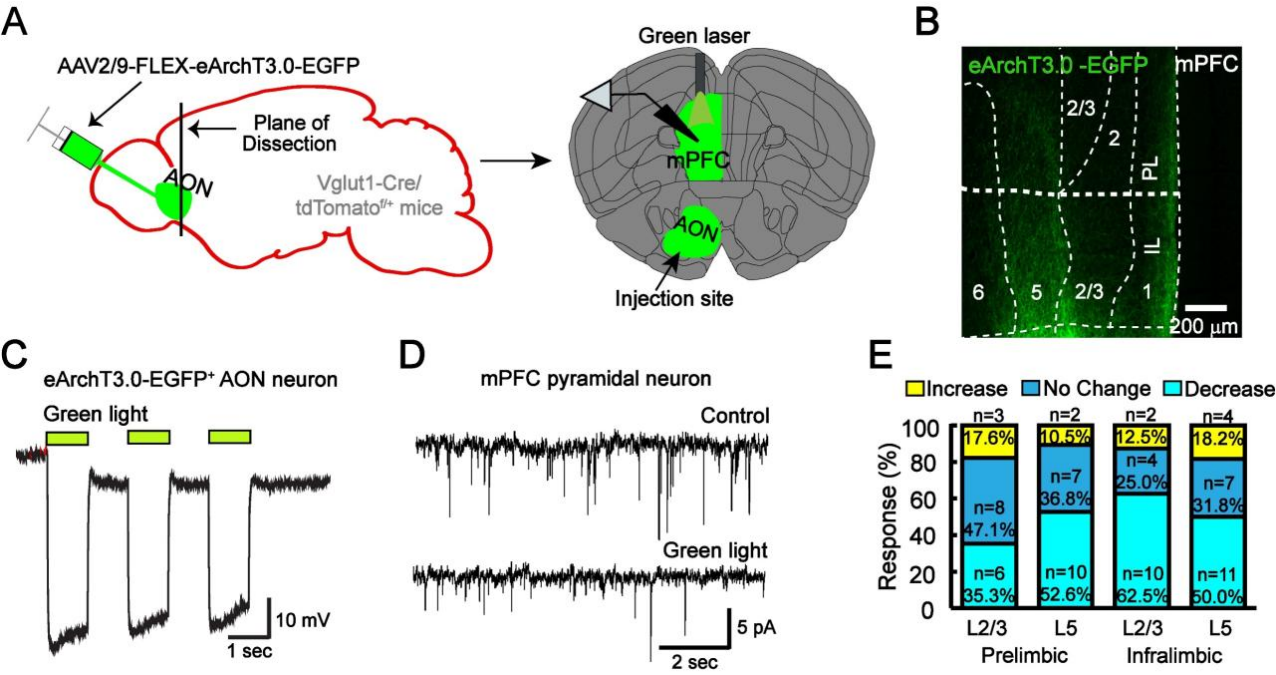
Optogenetic inhibition of AON neurons reduces sEPSC frequency in ~50% of mPFC pyramidal neurons. **(A)** Schematic of viral injection and patch-clamp recording combined with optogenetics in brain slices. **(B)** eArchT3-EGFP expressing axons of AON neurons in the mPFC. **(C)** Green light-evoked membrane hyperpolarization of an eArchT-EGFP^+^ AON neuron. The initial baseline membrane potential was at −60 mV. Similar results were obtained from 5 AON neurons. **(D)** Representative raw traces of sEPSCs in mPFC pyramidal neurons under control and green light inhibition of AON neuron terminals. Voltage-clamp mode with holding potential = −70 mV. **(E)** Summary of sEPSC frequency changes in mPFC pyramidal neurons upon green light stimulation compared to no light control. The changes (increase, no change, or decrease) were determined by Kolmogorov-Smirnov statistical analysis for each cell (p<0.05 was considered significant). Data were obtained from 6 mice.

We next addressed the question to what extent AON neurons contribute to synaptic inputs into mPFC pyramidal neurons using optogenetic inhibition in brain slices. Cre-dependent AAV2/9-FLEX-eArchT3.0-EGFP virus was unilaterally injected into the AON of *Vglut1*-Cre;*tdTomato*^f/+^ mice to drive eArchT expression in AON neurons (Fig. 4A, 4B). This mouse line was chosen because it allowed unambiguous identification of pyramidal neurons via expression of Vglut1-tdTomato^+^. Four weeks later, whole-cell patch clamp recording was performed in acute brain slices. In the eArchT-EGFP^+^ AON neurons, green light induced hyperpolarization as expected and no rebound action potentials were observed under our recording condition (Fig. 4C). In mPFC Vglut1-tdTomato^+^ pyramidal neurons, the frequency (but not the amplitude) of spontaneous EPSCs (sEPSCs) significantly changed upon optogenetic inhibition of AON neurons (Fig. 4D, 4E). Specifically, 50.0% (37 out of 74 neurons) of mPFC pyramidal neurons showed a decrease in the sEPSC frequency, while a much lower percentage (14.9% or 11 out of 74 neurons) showed an increase (Fig. 4E). Taken together, these findings reveal that AON neurons provide a constant, excitatory input into the mPFC.

### Activation of AON neurons in freely behaving mice

**Figure 5.**
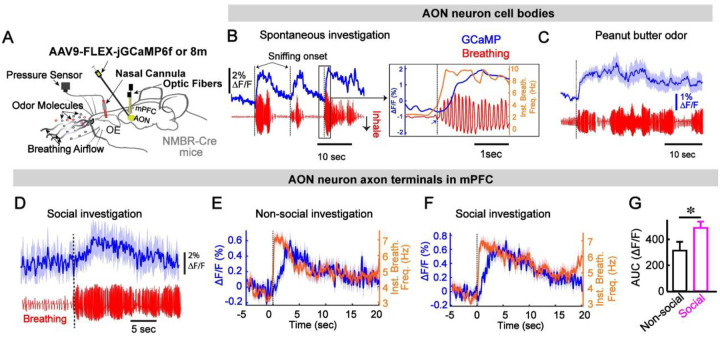
The AON→mPFC pathway is active during spontaneous and exploratory sniffing in freely behaving mice. **(A)** Schematic of viral injection and surgical implants: nasal cannula for breathing recording via a pressure sensor, optic fiber for GCaMP signal recording from AON neuron cell bodies or from their axon terminals in the mPFC. **(B)** GCaMP signals in AON neurons during spontaneous sniffing. Similar results from N=4 mice. Inset, expansion of traces inside the box together with the instantaneous breathing frequency curve. Blue arrow indicates sniffing onset coinciding with a sudden increase in instantaneous sniffing frequency. **(C)** GCaMP signals in AON neurons during investigation of peanut butter odor (9 trials from N=3 mice). **(D)** GCaMP signals from AON neuron axon terminals in the mPFC during social investigation (8 trials from N=4 mice). **(E, F)** GCaMP signals from AON neuron axon terminals in the mPFC (blue) and instantaneous breathing frequency (red) during non-social (E; 80 sniffing bouts) and social investigations (F; 78 sniffing bouts) from N=4 mice. **(G)** Area under the curve (AUC) was calculated from GCaMP ΔF/F traces for 10 sec from the sniffing onset. Data are shown as mean ± SEM with two-sided, unpaired t-test (*p < 0.05). In B-F, dotted vertical lines denote sniffing onsets. For group data (C-F), darker-colored traces represent the average, while lighter-colored traces indicate the SD.

We next asked when AON neurons are active in freely behaving mice using Ca^2+^-based fiber photometry. We injected Cre-dependent AAV9-FLEX-GCaMP6f or -GCaMP8m (see Methods for details) virus unilaterally into the AON of *Nmbr*-Cre mice and implanted an optical fiber either in the AON (recording from AON neuron cell bodies) or in the mPFC (recording from the axon terminals of AON neurons) (Fig. 5A). Meanwhile, a nasal cannula was implanted for monitoring breathing signal via a pressure sensor (Fig. 5A). After four weeks for GCaMP expression, we recorded temporally demodulated 490 nm and 405 nm fluorescent signals (Ca^2+^-dependent and -independent, respectively). Transient increases of ΔF/F were observed during spontaneous sniffing bouts and during odor or social investigation (Fig. 5B–D). Sniffing onset was defined when the instantaneous breathing frequency curve starts to rise (inset in Fig. 5B). Compared with GCaMP signals induced by non-social investigation (i.e., spontaneous sniffing bouts without a social partner), those induced by social investigation exhibited increased area under the curve (AUC) (Fig. 5E–G). These results suggest that AON neurons constantly provide excitatory input to the mPFC during exploration, with stronger input during social investigation.

### Contribution of the AON→mPFC pathway to social recognition

**Figure 6.**
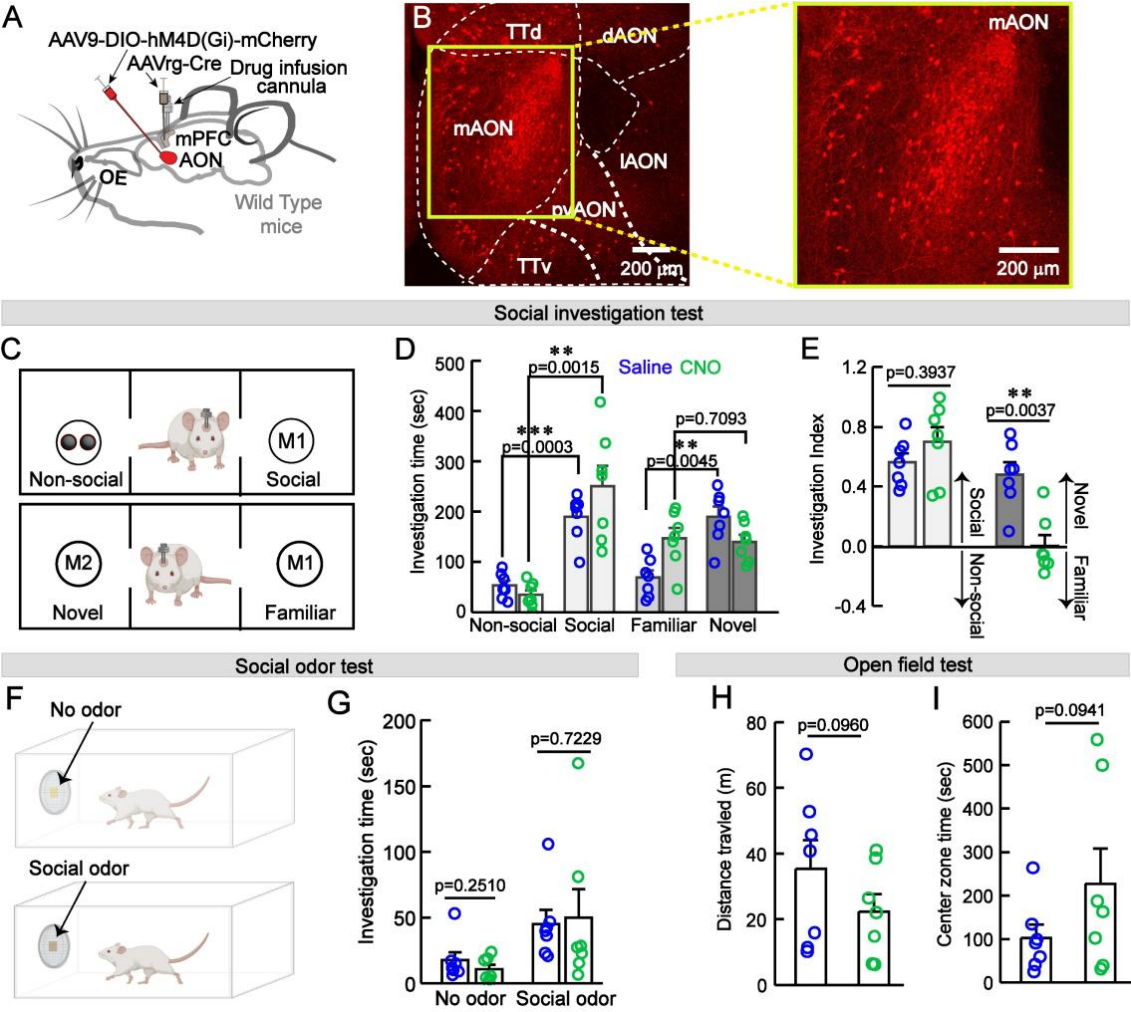
Chemogenetic inhibition of the AON→mPFC pathway impairs social recognition but not sociability. **(A)** Schematic of bilateral viral injection and cannula implantation for saline/CNO infusion into the mPFC. **(B)** Confocal image showing HM4Di-mCherry labeled mPFC-projecting AON neurons. d, dorsal. m, medial. l, lateral. pv, posteroventral. v, ventral. Similar results were obtained from 3 mice and verified in the mice used in behavioral tests. **(C)** Schematic of three chamber social investigation apparatus and the test paradigm. **(D, E)** Investigation time (mean ± SEM) (D) and investigation index (E) when saline or CNO (inhibition of AON neuron axon terminals in mPFC) was infused bilaterally into the mPFC. Investigation index is defined as the difference of investigation time between the two sides over the total investigation time of the two sides. **(F)** Schematic of no odor control and social odor test apparatus. **(G)** Investigation time (mean ± SEM) towards no odor (control) and social odor between saline and CNO infusion. **(H, I)** Open field test showing total distance traveled **(H)** and time spent in the center zone **(I)**. Data shown as mean ± SEM with two-sided, paired *t*-test, and significance denoted by * (p<0.05), ** (p<0.01), and *** (p<0.001).

To gain insight into the function of the AON→mPFC pathway, we utilized a chemogenetic inhibition approach (Fig. 6A). A retrograde AAVrg-EF1a-Cre virus was injected into the mPFC of wild-type mice to drive Cre expression in mPFC-projecting presynaptic neurons including those in the AON, and AAV9-DIO-hM4D(Gi)-mCherry, inhibitory DREADD, into the AON. The mPFC-projecting AON neurons were then verified by mCherry expression (Fig. 6B). A three-chamber test was used to assess sociability and social recognition (Fig. 6C). The test mouse was first released in the middle chamber for 5 min to minimize the novelty stress. Then the mouse was habituated in the entire arena for 10 min, and then it was released in the middle chamber, allowing 10 min free exploration of the two side chambers: one with a non-social object (a pair of marbles) and the other with a social object (a same-sex juvenile mouse M1). In the next 10 min, the non-social object was replaced by another same-sex juvenile mouse M2 (from a different home cage of M1) as the novel mouse and now M1 as the familiar mouse. In control condition (saline), the test mice spent more time investigating a social object (M1) than a non-social object (marble) (assessment for sociability). In the following session, the test mice spent more time investigating the novel (M2) than the familiar mouse (M1) (assessment for social recognition) (Fig. 6D, 6E).

Two approaches were used for chemogenetic inhibition. In the first set of experiments, Clozapine N-oxide (CNO at 3–5 mg/kg, a DREADD ligand) or saline was administrated systematically to inhibit mPFC-projecting AON neurons. CNO treatment impaired social recognition compared to saline control, but with no evident change in sociability (Supplemental Fig. S3A, B). Because mPFC-projecting AON neurons also project to other brain regions (Supplemental Fig. S4), we could not attribute the behavioral effect solely to the AON→mPFC pathway. In order to achieve specific inhibition of the AON→mPFC pathway, in a second set of experiment, we locally infused CNO (0.5 μl, 1 mM) or saline bilaterally into the mPFC. CNO infusion reduced social recognition but not sociability (Fig. 6D, E). In both sets of experiments, CNO did not significantly alter the mouse behavior in social odor investigation (volatile urine odor from the same sex juvenile mouse) and in the open field and compared to saline (Fig. 6F–H; Supplemental Fig. S3C–E). These findings suggest that inhibition of the AON→mPFC pathway specifically impairs social recognition without affecting sociability, and this effect cannot be explained by changes in social odor detection, locomotion, or anxiety level.

### Conserved AON–prefrontal functional connectivity in the human brain

**Figure 7.**
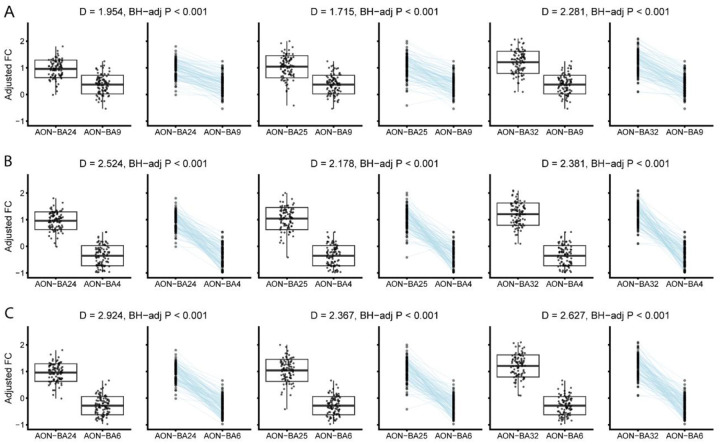
Comparison of functional connectivity (FC) between hAON–hACC and hAON–nearby frontal regions. (**A**) FC between hAON and hACC subregions (BA24, BA25, BA32) compared with FC between hAON and BA9 (dorsolateral prefrontal cortex). (**B**) FC between hAON–hACC and hAON–BA4 (primary motor cortex). (**C**) FC between hAON–hACC and hAON–BA6 (supplementary motor cortex). Functional connectivity (FC; Fisher z-transformed) was adjusted for age, sex, race, cigarette smoking status, and head motion. Two-sided paired t tests were used for comparisons. Adjusted FC values are shown as boxplots with overlaid individual data points, with paired observations connected by lines. Effect sizes (Cohen’s d) and Benjamini–Hochberg adjusted p values (correction for multiple comparisons) are indicated.

We asked the question of whether the human brain also has a strong AON-prefrontal connection, as observed in mice. We have processed resting-state (rs)-fMRI data from 93 healthy controls and measured the functional connectivity (FC) between the human AON (hAON) and human anterior cingulate cortex (hACC) Brodmann Area (BA) 24, 25, and 32 (equivalent to the mouse mPFC spanning the anterior cingulate, prelimbic and infralimbic cortices; see Discussion). Regions analyzed are shown Supplemental Fig. S5. Adjusted FC values were estimated using linear regression while controlling for potential confounders including age, sex, race, tobacco use, and head motion. De-identified demographic data for the study participants is provided in Supplemental Table S1. The hAON exhibits significantly higher connectivity strengths with hACC subregions than the dorsolateral prefrontal cortex BA9 (a dorsolateral PFC region crucial for higher brain functions), the nearby primary motor cortex BA4 and the supplementary motor cortex BA6 (Fig. 7A–C), indicating a specific functional connection between hAON and hACC subregions. Overall, these findings support the presence of strong network connections between the olfactory system and the prefrontal cortex in both mice and humans.

## Discussions

This study investigates a previously overlooked olfactory-prefrontal circuit that serves as a critical bridge between sensory processing and high-level social cognition. Using whole-brain circuit tracing, we demonstrate that olfactory cortical neurons in the AON selectively target the prefrontal cortex among the neocortices and the AON→mPFC pathway is required for social recognition. The identification of a homologous functional connectivity in the human brain underscores the significance of these findings.

Compared to other sensory systems, the olfactory system is unique with its tight connection with the prefrontal cortex. The projection neurons of the MOB transmit sensory information to the primary olfactory cortices, including the PIR and AON. Subsequently, the primary olfactory cortices directly project to the prefrontal cortex. The PIR sends massive projections to the orbitofrontal cortex (OFC, a pathway essential for smell perception^44–50^). The AON is physically proximate to the mPFC and provides a direct pathway linking the olfactory system to the mPFC (Fig. 2), consistent with previous tracing studies^13–16,51^. In addition to the direct AON→mPFC pathway, olfactory signals can be conveyed to the mPFC via alternative pathways^6^. For instance, there is a direct projection from the posterior PIR to the mPFC, a pathway required in social transmission of food safety information^52^. Additionally, the PIR can carry olfactory information to the mPFC via polysynaptic projections via the OFC, the mediodorsal thalamus, etc. Although both the AON and PIR receive direct input from the MOB, they exhibit distinct odor responses and likely serve different roles in processing social cues^12,53–56^. It is plausible that neurons in the AON and PIR convey complementary social information that is integrated within the mPFC. Interestingly, the PIR also receives social cues directly from the mPFC and sends feedback projections to the MOB to modulate social recognition^53^. In contrast, the AON→mPFC projection is unidirectional, i.e., the AON does not receive top-down projection from the mPFC (Fig. 2).

Consistent with the anatomical evidence for the AON→mPFC projection (Fig. 2), optogenetic activation of AON neurons elicited monosynaptic EPSCs in ~50% of mPFC pyrimdal neurons and interneurons (Fig. 3, Supplemental Fig. S2). Although mPFC neurons receive monosynpatic input from numerous brain regions^51^, it is intriguing that optogenetic inhibition of AON neurons reduced the frequency of sEPSCs in nearly 50% of mPFC pyramidal neurons (Fig. 4). The reported percentages should be considered as estimations attibuted to techincal caveats. On one hand, not all AON neurons expressed ChR2-EYFP or eArchT-EGFP, limited by the efficiency of AAV infection, potentially leading to underestimation. On the other hand, patch-clamp recordings were targeted to mPFC neurons near dense EYFP^+^ or EGFP^+^ axonal fibers of AON neurons, potentially leading to overestimation. Nevertheless, these findings reveal that AON neurons provide substantial synaptic input onto mPFC neurons in brain slices at the baseline condition. Social behaviors in rodents greatly depend on chemosensory cues, accompanied by modulation of breathing/sniffing patterns during odor/social investigation^37,38^. AON neurons and their axonal terminals in the mPFC exhibited robust activation during spontaneous sniffing and odor/social investigation (Fig. 5), supporting constant input from the AON to mPFC in freely behaving mice.

By receiving direct input from the MOB^6,8^, ventral hippocampus^9,10^, and hypothalamic oxytocinergic neurons^11,12^, the AON is positioned in the intersection between odor memory and social recognition. This study identifies the mPFC as the downstream target through which the AON exerts its role in social recognition. Chemogenetic inhibition of mPFC-projecting AON neurons (Supplemental Fig. S3) or more specifically, the AON→mPFC pathway (Fig. 6), impaired social recognition without interfering sociability. These findings lends further support to the notion that the mPFC and its associated circuitry play a crucial role in social cognition^17–19^. The mPFC contains partially overlapping neural ensembles that encode ongoing social interaction, social novelty, and conspecific identity in real time^20–23^. AON neurons as well as PIR neurons that are activated during odor and social investigation likely provide an important source for coactivation of a subset of mPFC neurons in adaptive cognitive behaviors^57^. This study adds to the literature that the understudied AON→mPFC pathway is required in social recognition, but not in sociability and general odor detection, highlighting dissociated functions of subpopulations of mPFC neurons. Although delineating the specific contributions of each olfactory cortical region (AON versus PIR) to social recognition will require further investigation, emerging evidence suggests that interactions between the olfactory system and the mPFC are essential for this process.

Each mPFC subdivision and its associated neural circuits serve distinct functions in social behaviors^17–19^. Since the AON is situated ventral to the mPFC, it is not surprising that the number of neurons receiving direct AON input was greater in the inframbic cortex (more ventral) than in the prelimbic cortex (Fig. 2). This trend was consistent with the patch-clamp analysis of monosynpatic connected neurons (Figs. 3, 4 and Supplemental Fig. S2). In addition, the *Nmbr*-Cre mice do not allow us to separate the AON from the tenia tecta in the tracing experiments because the principal neurons in both regions express *Nmbr* (Fig. 1). The dorsal tenia tecta and adjacent dorsal peduncular cortex modulate affective behavior in mice via projections to the hypothalamus^58^. Notably, the dorsal peduncular cortex and infralimbic area, both receiving direct AON input (Fig. 2), constitute visceromotor cortex that projects to hypothalamus and brainstem, regulating neuroendocrine, sympathetic, and parasympathetic output^59^. Further studies are needed to distinguish the contributions of the AON and tenia tecta, as well as those of mPFC subdivisions, in social cognition and related functions.

Compared to the mouse AON, it is technically challenging to identify the hAON due to its small size relative to the rest of the brain and its ventral location in the human brain^6^. A previous fMRI study from healthy subjects clusters the primary olfactory cortices based on resting-state, whole-brain functional connectivity patterns, leading to identification of the hAON^60^. The mouse mPFC has anatomically and functionally homologous structures across species including humans (hACC)^17,61,62^. By utilizing resting-state fMRI data from a large cohort of healthy controls, we established that the hAON maintains significantly stronger functional connectivity with subregions of the hACC (BA 24, 25, and 32) than with control regions including the dorsolateral prefrontal cortex (BA 9) or motor cortices (BA 4 and 6) (Fig. 7). This study indicates that the connectivity from the AON to mPFC (hACC) is a conserved feature across species. These conserved olfactory-prefrontal circuits may underlie the frequent co-occurrence of olfactory deficits, social deficits, and executive dysfunction observed in various psychiatric populations.

## Methods

### Animals

*Nmbr*-Cre mice were generated using CRISPR-CAS9 gene editing technique (see below). *Vglut1*-Cre (Vglut1-IRES2-Cre-D; JAX Stock No: 023527^63^) and Ai9;tdTomato^f/+^ (Rosa26-CAG-LSL-tdTomato-WPRE; JAX Stock No: 007905^64^) mice were purchased from the Jackson laboratory and bred with wildtype (C57BL/6J; JAX Stock No: 000664) mice. All mice were housed under a 12-hour light/dark cycle, with food and water available *ad libitum*, in a temperature- and humidity-controlled animal facility. All behavioral and recording procedures were performed during the light phase of the cycle. Both male and female mice (2–6 months old, unless otherwise stated) were used and data were combined as no sex differences were evident. All procedures were performed in accordance with NIH Guidelines for care and use of Laboratory Animals and were approved by Animal Care and Use Committee at the University of Pennsylvania.

### Viruses

The viruses used for retrograde transsynaptic tracing AAV5-CAG-DIO-loxP-TVA66T-2A-mCherry, AAV8-CAG-DIO-loxP-G, and (EnvA)-SAD-ΔG-EGFP were described in a previous study^65^. AAV(DJ/8)-EF1a-FLEX-synaptophysin::EGFP-WPRE-hGHpA^66^ and AAV2/9-CAG-DIO-eArchT3.0-EGFP^67^ were from the Neuroconnectivity Core, Baylor College of Medicine. All other viruses were from Addgene: AAV1-EF1a-double floxed-hChR2(H134R)-EYFP-WPRE-HGHpA (RRID:Addgene_20298), AAVrg-EF1a-Cre (RRID:Addgene_55636), AAV1-hSyn-Cre-WPRE-hGH (RRID:Addgene_105553), AAV9-hSyn-DIO-hM4D(Gi)-mCherry (RRID:Addgene_44362), AAV9-Syn-Flex-GCaMP6f-WPRE-SV40 (RRID:Addgene_100833), AAV9-syn-FLEX-jGCaMP8m-WPRE RRID:Addgene_162378), AAV9-hSyn-DIO-EGFP (RRID:Addgene-50457).

### Generation of *Nmbr*-Cre knock-in mice

Knock-in mice expressing P2A-Cre from the endogenous *Nmbr* gene locus were generated using CRISPR–Cas9-mediated homology-directed repair in fertilized zygotes^39,40^. A single guide RNA (crRNA: GCAAGAAATAGCACTGTGAT (mouse ES cell tested) and tracrRNA; IDT, Integrated DNA Technologies) was designed to target the last coding exon of the *Nmbr* gene, immediately upstream of the stop codon and adjacent to an N(G)GG PAM site. The guide RNA (crRNA and tracrRNA) was mixed with recombinant Cas9 protein (PNA Bio, CA, USA) prior to microinjection. A single-stranded oligodeoxynucleotide donor containing the in-frame P2A-Cre coding sequence was used as the repair template, flanked by 36 bp 5′ and 3′ homology arms corresponding to the *Nmbr* open reading frame in the C57BL/6 genome. Zygote microinjections were performed at the Johns Hopkins Transgenic Mouse Core following established protocols, and injected embryos were transferred into pseudopregnant recipient females. Genomic DNA was isolated from tail biopsies of live-born pups and screened by PCR for editing at the *Nmbr* locus. Junction-specific PCR spanning the 5′ and 3′ homology arms, as well as amplification across the full inserted sequence, was used to identify correctly targeted alleles. PCR products were validated by Sanger sequencing to confirm precise, in-frame insertion of the P2A-Cre cassette and preservation of the surrounding coding region. Founder mice carrying the correctly targeted allele were bred with wild-type C57BL/6 mice to assess germline transmission and with Ai9;tdTomato^f/+^ mice to evaluate Cre recombinase activity (Fig. 1B). A three-primer PCR assay was established for routine genotyping, allowing discrimination of wild-type, heterozygous, and homozygous *Nmbr*-P2A-Cre alleles based on product size.

### RNAscope multiplex fluorescent assay

For RNAscope in situ hybridization experiments, mice were deeply anesthetized with a ketamine/xylazine mixture (200 mg/20 mg/kg body weight). Brains were rapidly extracted, embedded in OCT compound and frozen using a dry ice-ethanol bath. Coronal brain sections (20 μm) containing the AON were cut on a cryostat, mounted onto Superfrost Plus microscope slides (Fisher, 22-034-979), and air-dried at room temperature for >2 hrs. All pre-hybridization procedures were conducted under RNase-free conditions. Double fluorescent *in situ* hybridization (FISH) was performed according to the RNAScope^®^ 2.0 Fluorescent Multiple Kit User Manual for Fresh Frozen Tissue^41^. RNAscope probes Mm-Nmbr-C2 (406461-C2), Mm-Slc17a7-C2 (416631-C2), tdTomato-C3 (317041-C3), were purchased from Advanced Cell Diagnostics (ACD).

### Stereotaxic surgery

For stereotaxic surgery, mice were anesthetized in an induction chamber with 3% isoflurane (vol/vol, mixed with oxygen) and then transferred to a stereotaxic frame (Model 940, David Kopf Instruments) equipped with a heating pad. Isoflurane was maintained at 1.5–2% via a nose cone for the remainder of the procedure. Body temperature was maintained at 37 °C using a temperature control system (TC-1000, CWE Inc.). A local anesthetic (bupivacaine, <2 mg/kg body weight, subcutaneous) was administered at the incision site prior to skin incision. Craniotomies for viral injection and cranial implantation were performed after leveling bregma and lambda in the horizontal plane and determining stereotaxic coordinates relative to bregma (based on^68^). For AON injection, the microinjection needle was angled to access the AON by passing through the MOB, targeting coordinates of +3.0 mm AP, +0.9 mm ML, and 3.5 mm DV. For mPFC injection, coordinates of +1.7–1.9 mm AP, +0.3 mm ML, and 2.5–2.7 mm DV were used.

### Circuit tracing

#### Retrograde transsynaptic tracing.

For rabies virus-mediated retrograde transsynaptic tracing, a mixture of AAV5-CAG-DIO-loxP-TVA66T-2A-mCherry and AAV8-CAG-DIO-loxP-G (1:1 ratio; total volume 300 nl) was unilaterally injected into the AON of *Nmbr*-Cre mice. Injections were performed using a 33-gauge Hamilton needle connected to an UltraMicroPump (WPI) mounted on a stereotaxic frame, with a delivery rate of 30 nl/min. The needle was left in place for 10–15 minutes after infusion to allow for diffusion before being slowly withdrawn. After a 10-day recovery period, EnvA-pseudotyped SAD-ΔG-EGFP rabies virus (300 nl) was injected into the same site to initiate transsynaptic labeling. Seven days later, mice were anesthetized and transcardially perfused with ice-cold PBS followed by 4% paraformaldehyde (PFA). Brains were collected, postfixed overnight at 4 °C, and then transferred to PBS. Coronal sections (100 μm) were prepared using a Leica VT1200S vibratome. Sections were sequentially incubated in glycerol/PBS solutions (50% 20 minutes, followed by 70% for 20 minutes), mounted onto glass slides, and imaged using a Leica SP5 confocal microscope with LAS AF Lite software and 473 nm, 599 nm, and 635 nm lasers (10× and 40× objectives) and fluorescent microscope (Leica DM5000 B). Starter cells were identified at the injection site by co-expression of mCherry and EGFP. Presynaptic input regions were mapped by identifying EGFP-positive, mCherry-negative cells across every other coronal section throughout the brain. Brain regions in coronal sections were identified based on the Allen Mouse Brain Atlas.

#### Anterograde tracing.

*Nmbr*-Cre-*tdTomato*^f/+^ mice received unilateral injections of 300–500 nl of AAV(DJ/8)-EF1a-FLEX-synaptophysin::EGFP-WPRE-hGHpA into the AON. In a subset of animals, Cre-dependent AAV1-DIO-ChR2-EYFP or AAV2/9-DIO-eArchT-EGFP (300–500 nl) was also used. Four weeks after injection, mice were perfused with ice-cold PBS followed by 4% PFA, and brains were collected, postfixed overnight at 4 °C, and sectioned coronally (100 μm) using a vibratome. Sections were mounted and imaged using a Leica SP5 confocal microscope with LAS AF Lite software and fluorescent microscope (Leica DM5000 B). For anterograde transsynaptic tracing of AON neurons, AAV1-Cre virus (300 nl) was unilaterally injected into the AON of *tdTomato*^f/+^ mice. Five weeks after injection, mice were perfused with ice-cold PBS followed by 4% PFA. Brains were collected, postfixed overnight at 4°C, and sectioned coronally (100 μm) using a vibratome. Sections were mounted and imaged as described above.

#### Anterograde transsynaptic tracing.

To label projection pattern of AON neurons projecting to the mPFC, AAVrg-Cre virus (300 nl) was unilaterally injected into the mPFC of wild-type mice, while a Cre-dependent AAV9-DIO-EGFP virus (300 nl) was injected into the AON to label retrogradely defined Cre-expressing neurons. Four weeks after viral injection, mice were perfused with cold PBS followed by 4% PFA. Brains were collected, postfixed overnight at 4°C, and sectioned coronally (100 μm) using a vibratome. Sections were mounted and imaged as described above.

### Patch-clamp recording and optogenetics

For patch clamp recording, acute brain slices were prepared from AAV-injected animals aged 3–6 months. Briefly, brains were rapidly removed and placed in ice-cold, oxygenated cutting solution containing 92 mM N-methyl-D-glucamine, 2.5 mM KCl, 1.2 mM NaH_2_PO_4_, 30 mM NaHCO_3_, 20 mM HEPES, 25 mM glucose, 5 mM sodium L-ascorbate, 2 mM thiourea, 3 mM sodium pyruvate, 10 mM MgSO_4_, and 0.5 mM CaCl_2_, continuously bubbled with 95% O_2_ and 5% CO_2_. Coronal sections (200 μm thick) were cut using a vibratome (Leica VT1200S) and transferred to artificial cerebrospinal fluid (aCSF) containing 126 mM NaCl, 2.5 mM KCl, 1.2 mM MgSO_4_, 2.4 mM CaCl_2_, 25 mM NaHCO_3_, 1.4 mM NaH_2_PO_4_, 11 mM glucose, and 0.6 mM sodium L-ascorbate, also continuously bubbled with 95% O_2_ and 5% CO_2_. Slices were incubated at 31 °C for 1 h and then maintained at room temperature for at least 30 min before recording. During recording, slices were transferred to a chamber continuously perfused with oxygenated aCSF. Recording pipettes (4–6 MΩ) were pulled from thin-walled borosilicate glass capillaries (inner diameter/outer diameter = 0.68/1.2 mm; 1B120F-4, WPI) using a horizontal Flaming/Brown puller (P-97; Sutter Instruments) and filled with an internal solution containing 120 mM potassium gluconate, 10 mM NaCl, 1 mM CaCl_2_, 10 mM EGTA, 10 mM HEPES, 5 mM Mg-ATP, 0.5 mM Na-GTP, and 10 mM phosphocreatine. Whole-cell patch-clamp recordings were acquired using an EPC-10 amplifier controlled by Pulse v8.74 (HEKA Elektronik) and analyzed with FitMaster (HEKA) and Mini Analysis (SynoSoft).

For optogenetic stimulation of AON-ChR2 fibers in the mPFC, single 1 ms blue-light pulses were delivered every 10 s using a pE-300^ultra^ (CoolLED) through a 40× objective. In voltage-clamp mode, cells were held at −70 mV. Light-evoked EPSCs were quantified as the difference between the mean baseline current before stimulation and the peak response. Response latency was defined as the first time point at which membrane current exceeded baseline + 2σ, where σ represented the standard deviation of the current during the 10–20 ms pre-stimulus window. Pharmacological tools included TTX (a sodium channel bloker), 4-AP (a potasium blocker), 6-Cyano-7-nitroquinoxaline-2,3-dione (CNQX; an AMPA receptor antagonist) and 5-phosphonopentanoic acid (AP5; an NMDA receptor antagonist). TTX was purchased from Tocris Bioscience, and all other drugs were purchased from Sigma Aldrich.

For optogenetic inhibition of AON-eArchT3.0-EGFP^+^ fibers in the mPFC, spontaneous excitatory postsynaptic currents (sEPSCs) were recorded in the absence and presence of green light (pE-300 ultra, CoolLED) through the 40× objective. sEPSC frequency and amplitude were analyzed to assess the effect of AON silencing on excitatory synaptic input to mPFC pyramidal neurons. For each cell, changes in sEPSC frequency during green light stimulation (increase, no change, or decrease) were assessed using the Kolmogorov–Smirnov test. Statistical significance was set at p < 0.05.

### Fiber photometry and nasal breathing recording

Ca^2+^-based fiber photometry and intranasal airflow recordings were performed simultaneously in the same cohort of mice. NMBR-Cre mice were prepared for both viral injection and nasal cannula implantation. AAV9-FLEX-GCaMP6f or GCaMP8m (300 nl) was injected unilaterally into the AON. A small hole was drilled in the nasal bone approximately 7.0 mm caudal to the tip of the nose on either the left or right side. The olfactory epithelium was carefully punctured to permit insertion of a nasal cannula (C311G/SPC, with dummy cannula C311DC; P1 Technologies) into the nasal cavity for respiration monitoring. Subsequently, a 400-μm optical fiber cannula was implanted above the injection site in the AON to record calcium signals from neuronal somata or in the mPFC to monitor activity from AON neuron axon terminals. Two stainless steel screws (Fine Science Tools) were placed bilaterally to stabilize the implants. All components, including the optical fiber and nasal cannula, were secured with 3M Vetbond adhesive (World Precision Instruments) and dental cement. Mice were allowed 4–5 weeks for recovery and for sufficient GCaMP expression. For fiber photometry, mice were connected to a 400-μm core optical fiber (0.48 NA; Thorlabs, M76L01) coupled to a four-port minicube (FMC4; Doric Lenses). Blue light (490 nm for GCaMP excitation; Thorlabs, M490F3) and violet light (405 nm for isosbestic control; Thorlabs, M405FP1) were delivered to the brain at 10–15 μW using an LED driver (Thorlabs, DC4104). Mice were tethered with the LEDs on for ~10 minutes prior to recording. Emitted fluorescence passed through a dichroic mirror and a 500–550 nm emission filter before detection by a femtowatt silicon photoreceiver (Newport, 2151). Signals were demodulated and recorded at 1017 Hz using an RZ5 processor and Synapse software (Tucker-Davis Technologies). Simultaneous behavioral videos (20 frames/sec) were acquired and synchronized in Synapse. Fluorescence changes (ΔF/F) were computed using the TDT MATLAB toolbox and custom MATLAB scripts. Data were down sampled to 113 Hz, normalized, and corrected by subtracting the 405-nm signal from the 490-nm signal. The resulting traces were then z-scored for further analysis. For breathing monitoring, the nasal cannula was connected to a sealed tubing system (C315CT; P1 Technologies) linked to a pressure sensor (CPXL04GF; Honeywell). Breathing signals were amplified using a DP-301 differential amplifier (Warner Instruments), bandpass filtered between 0.1–100 Hz, and acquired at 1017 Hz via an RZ5P processor (Tucker-Davis Technologies). Neural activity was inferred from GCaMP signals, while nasal airflow patterns were used to assess sniffing behavior as a measure of investigation.

The instantaneous breathing frequency curve was calculated from the breathing trace by detecting successive respiratory cycles. The time interval between consecutive inhalation peaks (inter-breath interval, IBI) was computed, and the instantaneous frequency (Hz) was obtained as the inverse of each interval (f = 1/IBI).

Experiments were conducted in the home cage or a similar open-field arena. Wire mesh cups, large enough to allow the stimulus mouse free movement, were placed within the chamber. On the test day, test mice were habituated for 5 minutes, followed by 5 minutes of exploration with empty cups. Social investigation was then assayed by placing an unfamiliar, same-sex juvenile mouse under a cup, after which the test mouse was allowed to explore for an additional 5 minutes. Investigation was defined as direct sniffing or oriented approach toward the cup accompanied by sniffing. GCaMP activity during spontaneous sniffing bouts longer than 5 seconds was quantified. Behavior was recorded from the side using a Logitech webcam (20 frames/sec) for offline analysis. For peanut butter odor presentation, a custom-made olfactometer was used. Air (40 cc) was passed through a tube containing peanut butter and delivered to the mice as an odor plume every 5 min for 2–3 trials, while simultaneously recording GCaMP signals and breathing, along with the odor presentation time stamp.

### Chemogenetic inhibition

For chemogenetic inhibition experiments, a retrograde AAVrg-EF1α-Cre virus (300–500 nl) was bilaterally injected into the mPFC, and a Cre-dependent AAV9-hSyn-DIO-hM4Di-mCherry virus (300 nl) was injected into the AON of wild-type mice to selectively inhibit mPFC-projecting AON neurons. In a subset of mice, bilateral drug cannulas (800-00219-00; RWD) with dummy cannulas (800-00249-00; RWD) and a metal cap (102-01133-00; RWD) were implanted above the mPFC for local infusion to permit local inhibition of AON neuron axon terminals. Mice were allowed to recover for 4–5 weeks to ensure sufficient viral expression. For chemogenetic inhibition, mice were randomly assigned to receive either Clozapine-N-oxide (CNO) or saline first, with treatment order reversed in subsequent sessions separated by a 2-week period. In mice without cannulas, CNO (3–5 mg/kg) or saline was administered intraperitoneally 20–30 minutes before behavioral testing. In mice with cannulas, 0.5 μl of either saline or CNO (1 mM) was infused bilaterally into the mPFC 20–30 minutes prior to testing using a Hamilton syringe connected to a catheter (901-03143-00; RWD) and bilateral internal cannulas (800-00294-00; RWD) inserted into the guide cannulas after removing the dummy cannulas and secured with a fixing screw (102-01129-00; RWD). After infusion, catheters were detached and behavioral assays were then conducted to assess the effects of neuronal inhibition. After experiments, mCherry fluorescence was examined to verify viral expression and targeting within the AON.

### Behavioral tests

All behavioral procedures were conducted during the light cycle (9:00–15:00). Experimental mice were transferred to the testing room at least 1 hour prior to testing to acclimate to the environment. Mouse behavior was recorded via a Logitech webcam (20 frames/sec) and analyzed offline using ANY-maze software for open field test or Behavioral Observation Research Interactive Software (BORIS; https://www.boris.unito.it/) for other tests.

#### Three-chamber social investigation test.

This test was used to assess sociability and social recognition. The apparatus consisted of three interconnected chambers (20 × 40 cm each), separated by removable doors. Each of the two wire mesh cups (large enough to allow the enclosed stimulus mouse to move and turn freely) was positioned in a side chamber to hold either a social stimulus or a non-social object. On the test day, the test mouse was first habituated in the middle chamber for 5 minutes with side chambers blocked. The mouse was then allowed to explore all three chambers for 10 minutes with empty wire mesh cups in the side chambers. Sociability was then tested by placing a pair of marbles (non-social object) under one cup and an unfamiliar, same-sex juvenile mouse (M1; social stimulus) under the other. The test mouse was released into the middle chamber and allowed to explore freely for 10 minutes. The investigation time (defined as direct sniffing or close orientation toward the cup) was quantified. Social recognition was assessed by replacing the marbles with a novel, unfamiliar same-sex juvenile mouse (M2) while retaining the familiar mouse (M1). The test mouse was again allowed to explore for 10 minutes, and the investigation time for the familiar (M1) and novel (M2) mouse was quantified.

#### Social odor presentation test.

Odor investigation was assessed in the home cage (19 × 29 cm) to minimize novelty stress. A 2 × 2 cm piece of filter paper was saturated with ~100 μl saline or fresh urine from same-sex juvenile mice and secured in a Petri dish beneath tightly meshed cloth (preventing direct contact) using a thick rubber band around the rim. The dish was attached to the center of the cage wall (19 cm side), ~2.5 cm above the floor. Mice were first allowed to explore the empty home cage for 2 minutes, followed by 5-minute exposures sequentially to the saline (no odor) and urine (social odor) stimuli, with the mouse gently confined to one half of the cage using a cardboard barrier during stimulus changes. Investigation was defined as direct sniffing toward the stimulus, and the total investigation time was quantified for each condition.

#### Open field test.

Locomotion and anxiety-level were assessed in a square open-field arena (40 × 40 × 40 cm). Each mouse was placed in the arena center and allowed to explore freely for 15 minutes. The arena was virtually divided into the central (25% of the total area) and peripheral zones. The apparatus was cleaned with 70% ethanol and dried between trials to remove residual odors. Testing occurred during the light phase under consistent lighting and ambient conditions.

### Functional connectivity analysis of human fMRI data

#### Participants.

This study utilized data from an established cohort at the Johns Hopkins Schizophrenia Center^69–72^. The study protocol was approved by the Johns Hopkins Medicine Institutional Review Board, and all individuals provided written informed consent prior to participation. Ninety-three healthy volunteers were enrolled through recruitment both within Johns Hopkins Hospital and from the surrounding community. Eligible participants were between 13 and 35 years of age and free of major medical and neuropsychiatric conditions. Exclusion criteria included a history of traumatic brain injury, malignancy, bleeding disorders, viral infection, neurological disorders, or intellectual disability. Individuals with a history of substance or alcohol abuse within the preceding three years were excluded. Participants reporting illicit drug use within the past two months were also excluded.

#### MRI Data Acquisition.

Imaging data were collected on a Philips dStream Achieva 3.0 Tesla MRI system. Resting-state functional MRI (rs-fMRI) and high-resolution structural images covering the cerebral cortex and subcortical gray matter (mean brain volume ≈1254.2 mL) were acquired during the same session. During rs-fMRI acquisition, participants were instructed to keep their eyes closed, remain awake, and minimize movement; compliance was monitored via an in-scanner camera.

Functional images were obtained using an axial acquisition scheme with a matrix size of 80 × 80, 36 slices, and a voxel resolution of 3 × 3 × 4 mm3. Imaging parameters included a repetition time (TR) of 2000 ms, echo time (TE) of 30 ms, and 210 volumes per run. Structural T1-weighted images were acquired in the sagittal plane with a matrix size of 170 × 170, 256 slices, and a voxel size of 1 × 1 × 1.2 mm3, using a TR of 6700 ms and a TE of 3.1 ms.

#### fMRI Preprocessing.

Resting-state fMRI data were preprocessed using Statistical Parametric Mapping (SPM12; Wellcome Centre for Human Neuroimaging, London, UK) implemented in MATLAB R2024 (MathWorks, Natick, MA, USA). The initial 10 volumes were discarded to allow for signal stabilization.

Motion correction was performed using a six-parameter rigid-body transformation to account for head movement, with motion estimates retained for downstream analyses. Slice timing correction was applied to adjust for inter-slice acquisition delays. Functional images were then normalized to the Montreal Neurological Institute (MNI) template space via nonlinear transformation and resampled to 3 × 3 × 3 mm3 isotropic resolution. Spatial smoothing was conducted with a Gaussian kernel of 6 mm full width at half maximum (FWHM) to enhance signal-to-noise characteristics and mitigate anatomical variability across participants.

#### Functional Connectivity Analysis.

Regions of interest (ROIs) included the anterior olfactory nucleus (AON) and Brodmann areas BA24, BA25, BA32, BA9, BA4, and BA6. Brodmann area masks were obtained from the Brodmann atlas distributed with MRIcron^73–75^. The AON ROI was defined based on a previously published diffusion MRI study characterizing the microstructure and striae of the human olfactory tract (cite: https://pubmed.ncbi.nlm.nih.gov/34759031/).

For each subject, mean blood oxygen level–dependent (BOLD) time series were extracted from each ROI. Functional connectivity (FC) was estimated as Pearson correlation coefficients between ROI time series and subsequently transformed using Fisher’s z-transformation.

### Statistical analysis

For mouse data, statistical tests (two-sided, unpaired or paired *t* and Kolmogorov-Smirnov) were performed using OriginPro 2022 (Origin Lab). For human data, all statistical analyses (two-sided, paired *t* test with Benjamini-Hochberg procedure to correct for multiple comparisons) were conducted using R (version 4.5.1).

## Supplementary Material

1
