# Supplementary material for: An olfactory-prefrontal cortical circuit supports social recognition
Source: Res Sq. 2026 Jun 1:rs.3.rs-9613537. Preprint. [Version 1] doi: 10.21203/rs.3.rs-9613537/v1 (PMC13308722; doi:10.21203/rs.3.rs-9613537/v1)
Supplement: 1 [file NIHPPRS9613537V1-supplement-1.pdf]

Supplemental Figures and Table

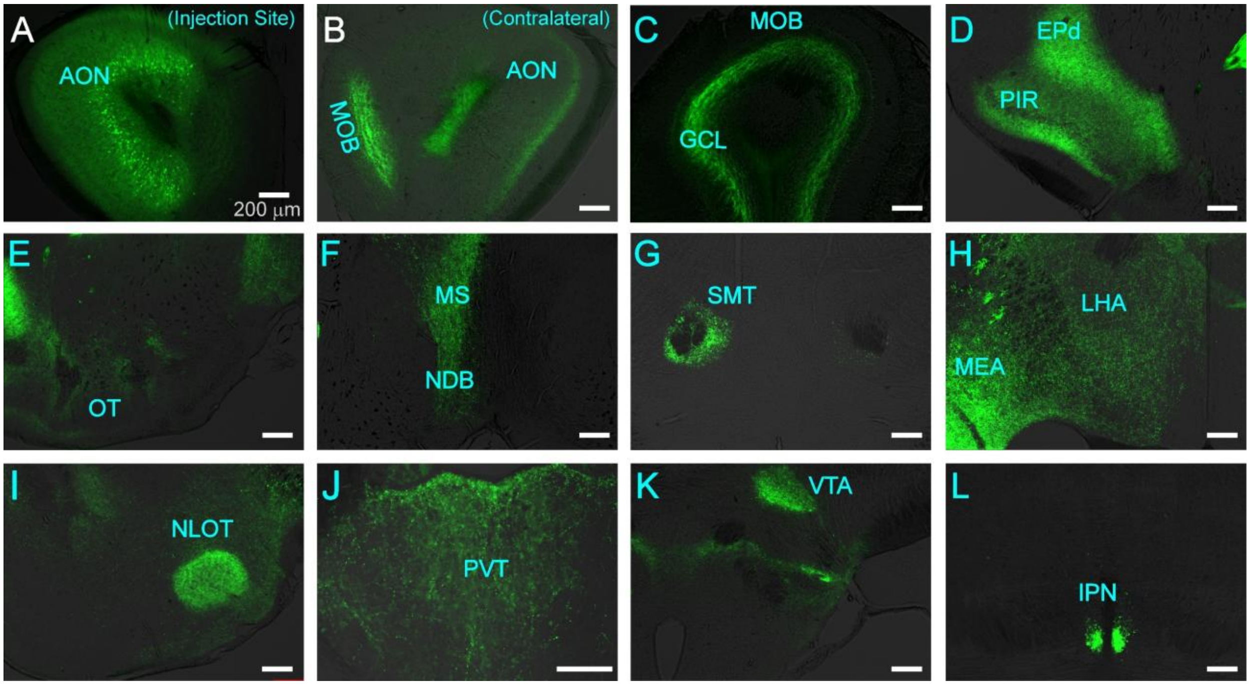

Supplemental Figure S1 (related to Fig. 2). Whole-brain tracing of synaptic outputs of AON neurons. (A) Confocal image showing EGFP expression at the injection site in the AON. **(B-L)** AON neuron axonal terminals (synaptophysin-EGFP$^+$ puncta) were observed in various brain regions, including contralateral AON (B); granule cell layer in the MOB (C); piriform cortex (PIR) and dorsal endopiriform nucleus (EPd) (D); olfactory tubercle (OT) (E); medial septum (MS) and nucleus of the diagonal band (NDB) (F); submedial nucleus of the thalamus (SMT) (G); lateral hypothalamus (LHA) and medial amygdalar nucleus (MEA) (H); nucleus of the lateral olfactory tract (NLOT) (I); paraventricular nucleus of the thalamus (PVT) (J); ventral tegmental area (VTA) (K); and interpeduncular nucleus (IPN) (L). Similar results were obtained from 5 mice. All scale bars = 200 µm.

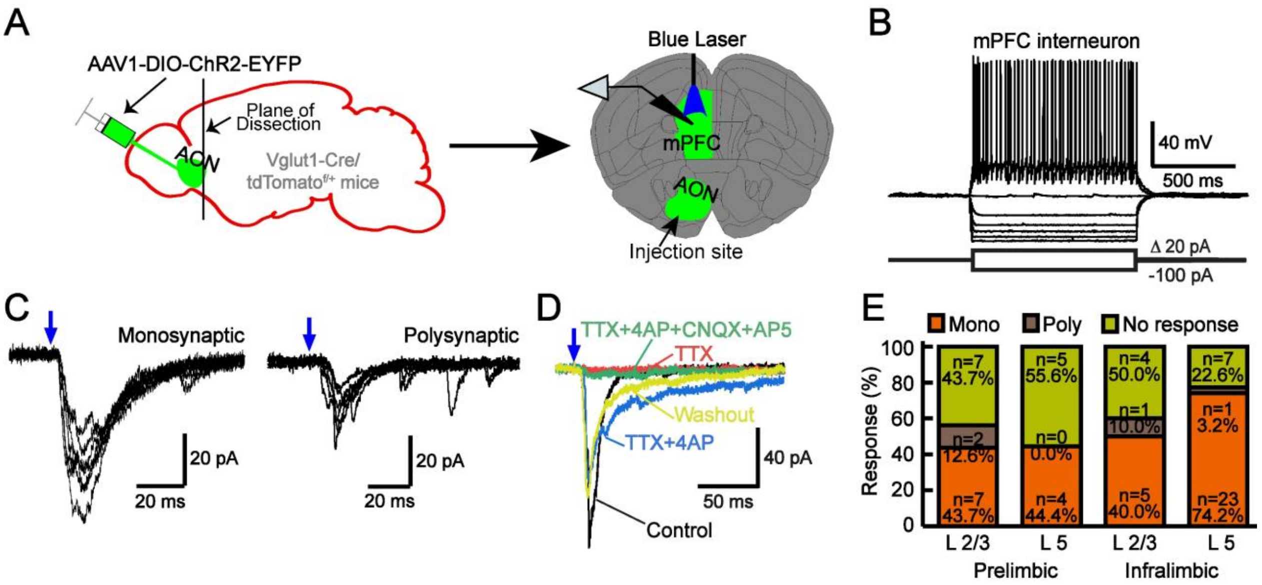

Supplemental Figure S2 (related to Fig. 3). AON neurons make excitatory synaptic connections onto mPFC interneurons. (A) Schematic of viral injection and patch-clamp recording combined with optogenetics in slices. **(B)** Typical firing pattern of mPFC interneurons. The baseline membrane potential was kept at -70 mV under current-clamp mode. **(C)** Blue light stimulation of ChR2 labeled fibers of AON neurons in mPFC induced mono- and poly-synaptic excitatory postsynaptic currents on mPFC interneurons. **(D)** Light evoked responses with short latency were blocked by TTX (1 μM), revived by TTX+4AP (1 mM), and further blocked by glutamate receptor antagonists CNQX (20 μM)+AP5 (50 μM). Similar results were obtained from 5 cells. **(E)** Summary of synaptic responses in L2/3 and L5 prelimbic and infralimbic pyramidal neurons upon blue light activation of AON neuron axons. Voltage-clamp mode with holding potential = -70 mV. Light pulse = 1 ms. Data were obtained from 7 mice.

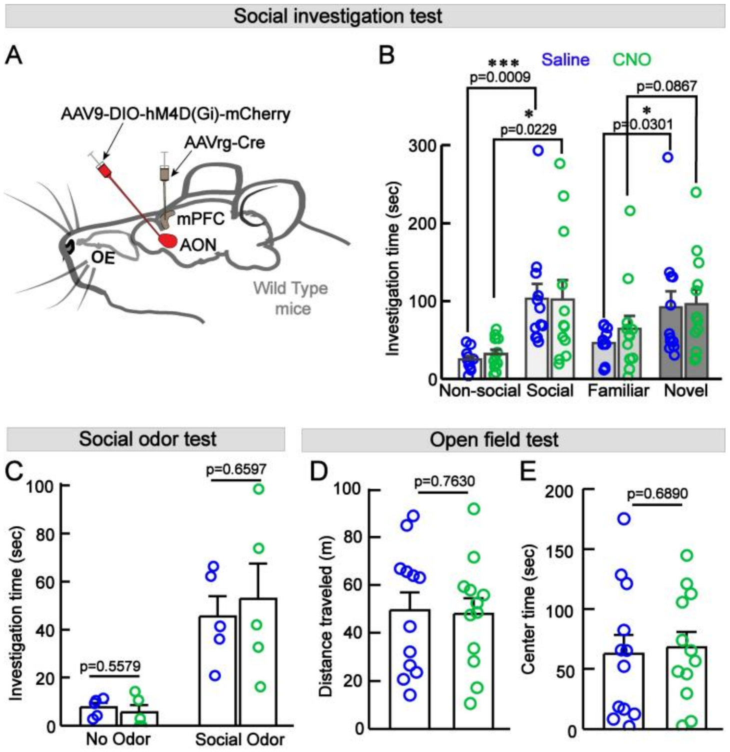

Supplemental Figure S3 (related to Fig. 6). Behavioral effects of chemogenetic inhibition of mPFC-projecting AON neurons via systematic administration of CNO. (A) Schematic of bilateral viral injection in the mPFC and AON. **(B)** Investigation time (mean ± SEM) following systemic administration of saline or CNO (to inhibit mPFC-projecting AON neurons). **(C)** Investigation time (mean ± SEM) towards no odor (control) and social odor between saline and CNO. (**D, E**) Open field test showing total distance traveled **(D)** and time spent in the center zone **(E)**. Two-sided, paired t test, and significance denoted by * (p<0.05) and *** (p<0.001).

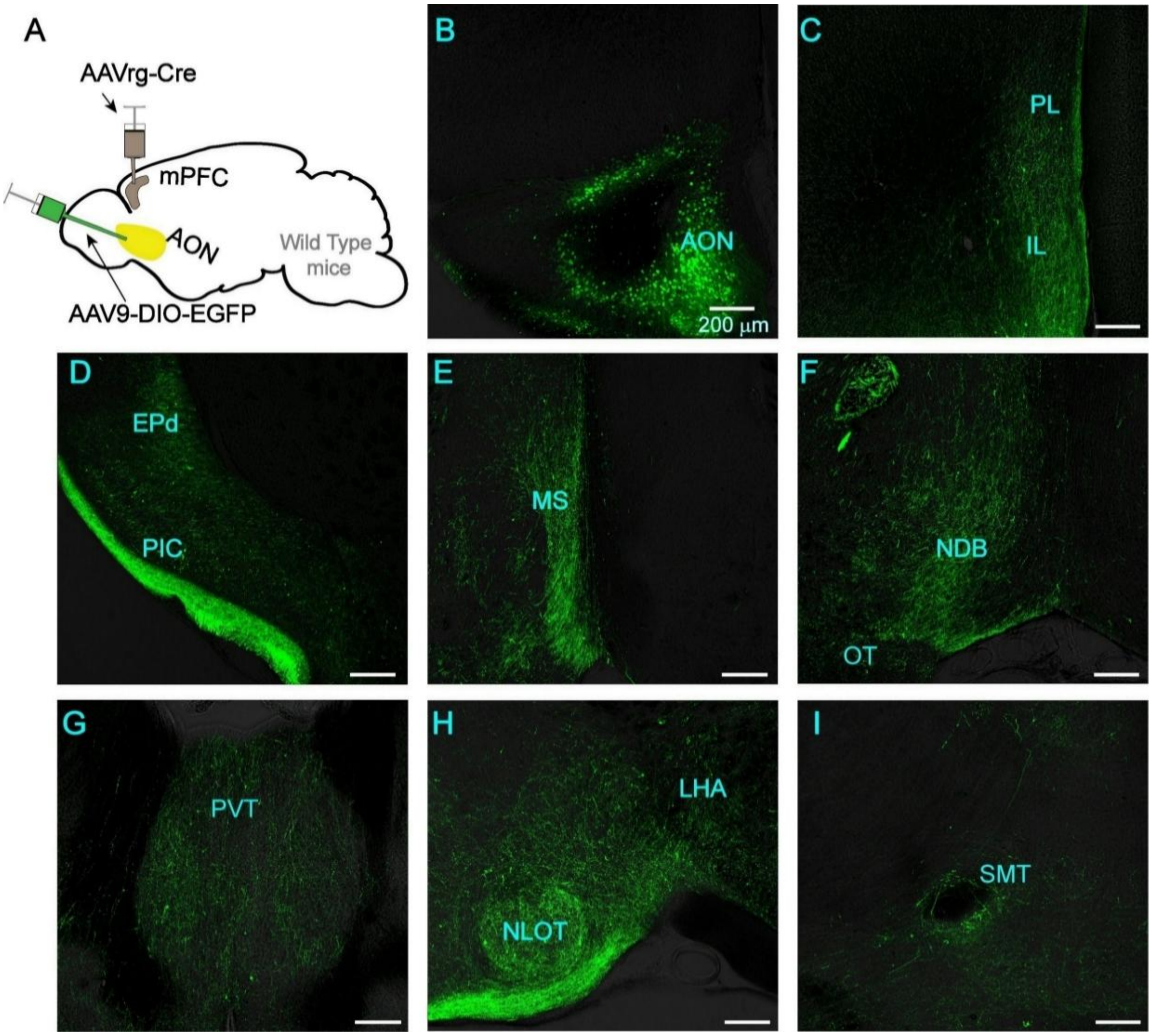

Supplemental Figure S4 (related to Fig. 6). The mPFC-projecting AON neurons also send axonal collaterals to other brain regions. (A) Schematic of viral injection in the mPFC and AON. **(B)** Confocal image showing EGFP expression in mPFC-projecting AON neurons. (**C-I**) EGFP+ axons of AON neurons were observed in various brain regions, including mPFC (C); piriform cortex (PIR) and dorsal endopiriform nucleus (EPd) (D)**;** medial septum (MS) (E); olfactory tubercle (OT) and nucleus of the diagonal band (NDB) (F); paraventricular nucleus of the thalamus (PVT) (G); lateral hypothalamus (LHA) and nucleus of the lateral olfactory tract (NLOT) (H); and submedial nucleus of the thalamus (SMT) (I).

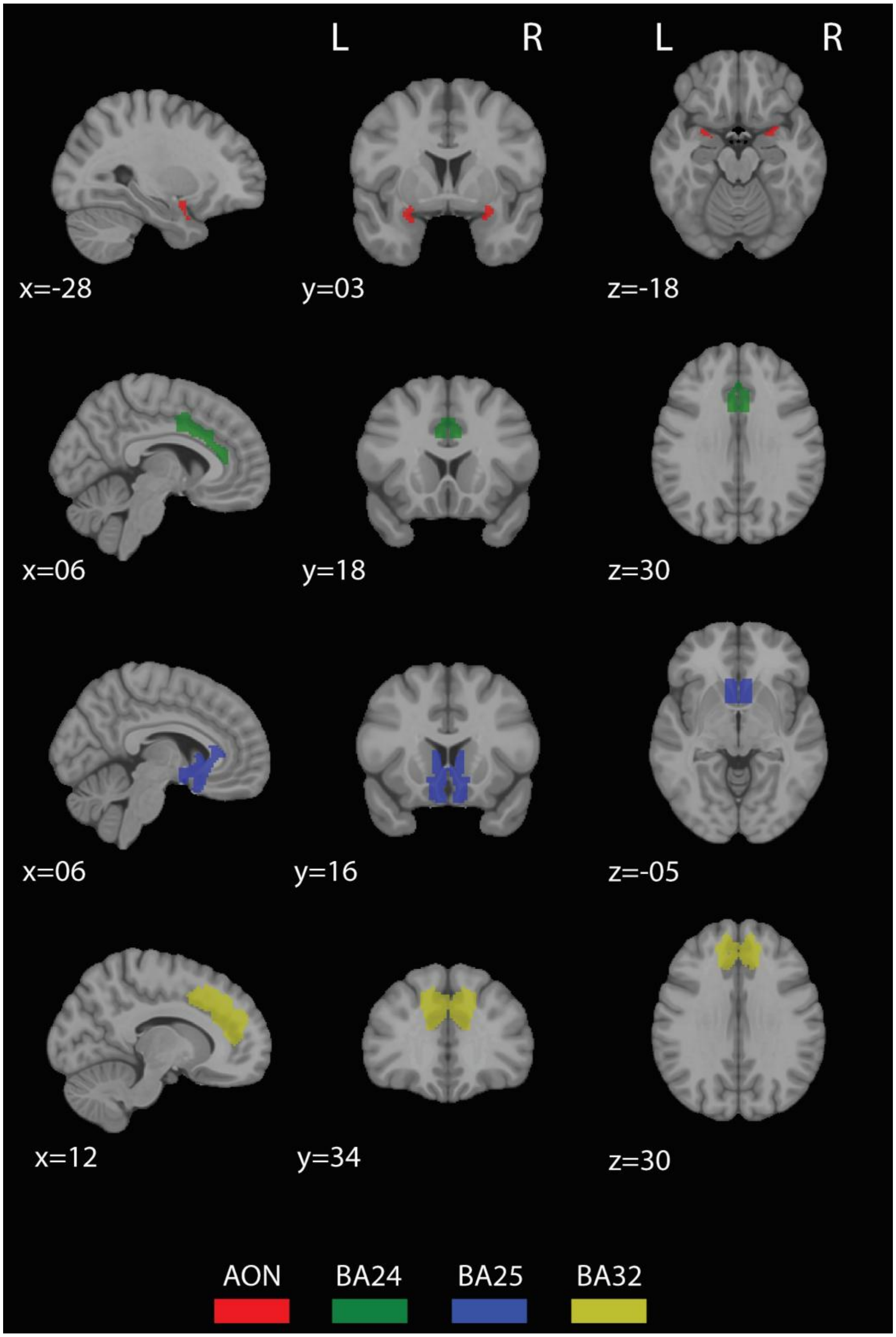

Supplemental Figure S5 (related to Fig. 7). Regions of interest in human brain fMRI. Anterior olfactory nucleus (AON) and anterior cingulate cortex (ACC; BA24, BA25, BA32) are shown overlaid on the MNI152 template. ROIs were defined in MNI space and visualized at 2 mm isotropic resolution.

Characteristic	Healthy Controls (N = 93)
Age, years	23.4 (3.9)
Sex, n (%)	
Male	41 (44.1)
Female	52 (55.9)
Race, n (%)	
Caucasian	27 (29.0)
African American	58 (62.4)
Asian	2 (2.2)
Other	6 (6.4)
Smoking status, n (%)	
No	90 (96.8)
Yes	3 (3.2)

Supplemental Table S1 (related to Fig. 7). Demographic data on human subjects.
